# Gli Activity Is Critical at Multiple Stages of Embryonic Mammary and Nipple Development

**DOI:** 10.1371/journal.pone.0079845

**Published:** 2013-11-18

**Authors:** Anupama Chandramouli, Sarah J. Hatsell, Alicia Pinderhughes, Lisa Koetz, Pamela Cowin

**Affiliations:** 1 Department of Cell Biology, New York University School of Medicine, New York, New York, United States of America; 2 The Ronald O. Perelman Department of Dermatology, New York University School of Medicine, New York, New York, United States of America; Cincinnati Children’s Hospital Medical Center, United States of America

## Abstract

Gli3 is a transcriptional regulator of Hedgehog (Hh) signaling that functions as a repressor (Gli3^R^) or activator (Gli3^A^) depending upon cellular context. Previously, we have shown that Gli3^R^ is required for the formation of mammary placodes #3 and #5. Here, we report that this early loss of Gli3 results in abnormal patterning of two critical regulators: *Bmp4* and *Tbx3*, within the presumptive mammary rudiment (MR) #3 zone. We also show that Gli3 loss leads to failure to maintain mammary mesenchyme specification and loss of epithelial Wnt signaling, which impairs the later development of remaining MRs: MR#2 showed profound evagination and ectopic hairs formed within the presumptive areola; MR#4 showed mild invagination defects and males showed inappropriate retention of mammary buds in Gli3^xt/xt^ mice. Importantly, mice genetically manipulated to misactivate Hh signaling displayed the same phenotypic spectrum demonstrating that the repressor function of Gli3^R^ is essential during multiple stages of mammary development. In contrast, positive Hh signaling occurs during nipple development in a mesenchymal cuff around the lactiferous duct and in muscle cells of the nipple sphincter. Collectively, these data show that repression of Hh signaling by Gli3^R^ is critical for early placodal patterning and later mammary mesenchyme specification whereas positive Hh signaling occurs during nipple development.

## Introduction

Mammary development becomes apparent in mice around E10.5 with expression of *Wnt10b* in mammary lines between the fore- and hind-limbs and in axillary and inguinal streaks [1], [2], [3]. Between E10.5 and E11.5, influx of epithelial cells towards and along these mammary lines and streaks leads to the formation of five pairs of placodes [4]. Analyses of knock-out mice and of human syndromes involving loss of mammary rudiments (MRs) or abnormal nipple number have identified more than a dozen factors essential for early mammary placodal development [3], [5], [6], [7], [8], [9], [10], [11]. Among these factors, MR#3 formation depends upon reciprocal antagonism between ventrally expressed Bmp4 and dorsal Tbx3 [12]. At ∼E12.5 the placodes form elevated buds. These buds sink below the periderm ∼E13.5 to form bulb-like structures, which induce underlying fibroblasts to become mammary mesenchyme [13], [14]. In females proliferation beginning at E15–E16, causes the mammary bulbs to sprout, penetrate the underlying developing fat-pad, and branch to form a small ductal tree (∼E18) [11], [13], [14], [15], [16]. The mammary mesenchyme in turn signals to the overlying epidermis to suppress hair follicles and form the nipple sheath [11], [13], [14], [15], [16]. In male embryos, intrinsic androgen response within the mesenchyme leads to atrophy of the buds [3], [9], [17], .

The Hedgehog (Hh) pathway plays a central role in the patterning and proliferation of many tissues, and its requirement in epidermal appendages, such as hair follicles and teeth, has been particularly well documented [21], [22], [23], [24], [25]. The mammalian Hh ligands, Sonic (Shh), Indian (Ihh) and Desert (Dhh), bind to twelve-pass transmembrane receptors Patched (Ptch1, Ptch2) on neighboring cells [26], [27]. This event relieves the seven-pass transmembrane protein smoothened (Smo) from Ptch-mediated repression and generates signals that are transduced by the Gli family of transcriptional activators and repressors (Gli1-3) [27], [28], [29]. Downstream Gli target genes, Ptch and Hhip (Hedgehog interacting protein), together with molecules acting at the level of ligand-binding such as cell surface bound Ig/fibronectin family members Cdo and Boc provide feedback mechanisms at various levels to keep the pathway in check [30]. Hh target gene expression is determined by the ratio of activator to repressor (Gli^A^: Gli^R^) forms of Gli proteins [31], [32], [33]. Gli2 is expressed independently of Hh signals in a functionally inactive form (Gli2^R^) but becomes processed in response to Hh signals into an activator (Gli2^A^) that initiates Hh target gene transcription [34], [35], [36], [37]. Gli1 is transcribed in a strictly Hh-dependent manner and once expressed, constitutively activates Hh target genes, including itself, and is considered to be a non-essential pathway amplifier [38], [39], [40], [41], [42]. These features make it a useful and reliable indicator of Hh pathway activation [31], [37]. Gli3 functions as a transcriptional activator (Gli3^A^) or repressor (Gli3^R^) depending on the cellular context [31], [43]. In the absence of Hh signals, Gli3^A^ is proteolytically processed into Gli3^R^. Hh signals prevent this proteolytic conversion and also transcriptionally downregulate *Gli3* [27], [35], [43], [44], [45]. Most tissues maintain a specific Gli^R^:Gli^A^ ratio by feedback mechanisms regulating downstream target gene expression. The processing of Gli proteins occurs within the primitive vestigial organelle, primary cilium [46]. Intraflagellar transport proteins (Ift) associate with kinesins or dyneins and are responsible for the formation and maintenance of primary cilia [47], [48].

Although hair follicles and mammary glands share many local inductive pathways, these appendages undergo strikingly different responses to Hh signaling [21], [22], [24], [49], [50], [51], [52], [53]. Hair and teeth require Hh signaling for downgrowth. We have shown that Gli3-mediated repression of Hh signaling is essential for the formation of MR#3 and #5 [51]. Loss of Gli3 exerts milder effects on the development of remaining MRs. However the molecular consequences of Gli3 action and whether it functions as an activator or repressor of Hh signaling or via Hh-independent functions at later stages have not been addressed.

Here we have investigated the effects of *Gli3* inactivation on factors involved early in the specification of MR#3 and found that Gli3 is required for the correct patterning of *Bmp4* and *Tbx3.* We further show that later in embryonic mammary development Gli3 loss or genetic misactivation of Hh signaling produce the same phenotypic spectrum of abnormal bud evagination, hair follicle encroachment and loss of sexual dimorphism. These data provide genetic evidence that repression of Hh signaling by Gli3^R^ is required for MR#2 invagination, hair follicle suppression, and loss of male mammary glands. Our results show that although mesenchymal Wnt signaling is activated in the absence of Gli3, later aspects of mammary mesenchymal specification are impaired and estrogen signaling and epithelial Wnt signaling fails. Finally, we show that positive Hh signaling is induced within specialized mesenchymal cell populations surrounding the lactiferous duct and is dynamically regulated within the smooth muscle cells of the nipple sphincter during the reproductive cycle.

## Results

In our experiments below we utilized a number of genetic approaches to define the function of Gli3 during mammary development. First we examined Gli3 extra-toes mutant mice (*Gli3^xt/xt^*) that lack Gli3 expression. To test whether the Gli3 phenotype results from loss of Gli3 repressor (Gli3^R^) or activator (Gli3^A^) function we utilize two strains. Gli1 strictly dependent on Hh signals for its expression and thus is an excellent reporter of positive Hh signaling [31], [37]. Therefore Gli1-LacZ reporter expression indicates where positive Hh signaling is activated. Although Gli2 initiates Hh signaling it is present prior to this event in an inactive or weakly repressive state. In contrast, Gli1, lacks any repressor domain or function and once expressed is a strong amplifier of the pathway [40], [41]. Thus, driving expression of the constitutive *Gli1* activator under the control of the *Gli2* promoter (*Gli2^1ki/+^* or *Gli2^1ki/1ki^*) tests the effect of activating Hh signaling within the Gli2 field of expression. If misactivation (*Gli2^1ki/1ki^* mice) produces the same or exacerbates the phenotypes of *Gli3^xt/xt^* mice, this provides genetic proof that the Gli3^xt/xt^ phenotypes result from loss of Gli3^R^ activity. In contrast, if the *Gli2^1ki/1ki^* and *Gli3^xt/xt^* phenotypes differ then Gli3 could be acting as a transcriptional activator of Hh signaling or in a manner independent of its role in Hh signaling.

### *Bmp4* and *Tbx3* Expression is Distorted within the Presumptive Mammary Placode #3 Region in *Gli3^xt/xt^* Embryos

**Figure 1:**
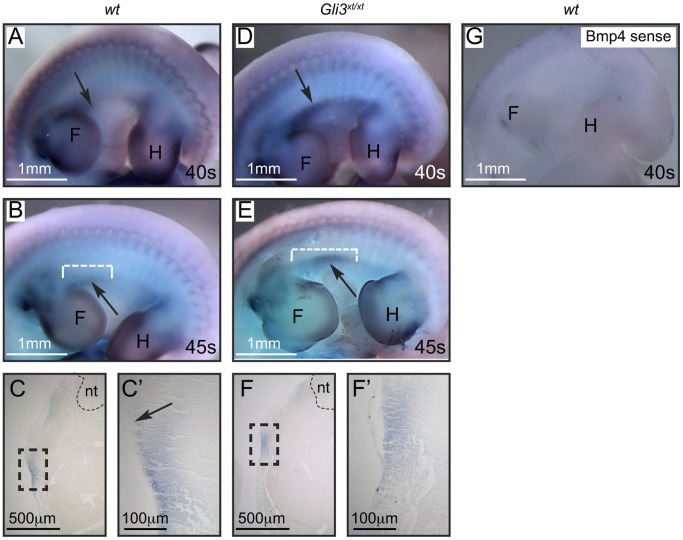
*Bmp4* mRNA expression is displaced in *Gli3^xt/xt^* embryos. Whole-mount *in situ* hybridization for *Bmp4* in wt (A, B) and *Gli3^xt/xt^* (D, E) embryos. In 40-somite stage wt embryos (A) *Bmp4* is expressed ventrally. This expression is more intense in *Gli3^xt/xt^* embryos at this stage (D). At the 45-somite stage, expression is reduced in wt embryos (B arrow), but expression is displaced dorsally and centrally into the locale of the developing mammary placode #3 in *Gli3^xt/xt^* embryos (E arrow). Distance from the axilla to the most distal point of *Bmp4* expression (B, E white dotted lines) was measured in mm. Sections through a 45-somite wt embryo (C) and higher power (C’) show mesenchymal expression ventral to the developing placode #3 (arrow). Sections through a 45-somite *Gli3^xt/xt^* embryo (F) and higher power (F’) show the displaced *Bmp4* expression. Control *in situ* hybridization with *Bmp4* sense probe is shown on a wt embryo in (G). Abbreviations: F – fore-limb, H – hind-limb, nt – neural tube, s – somite.

**Figure 2:**
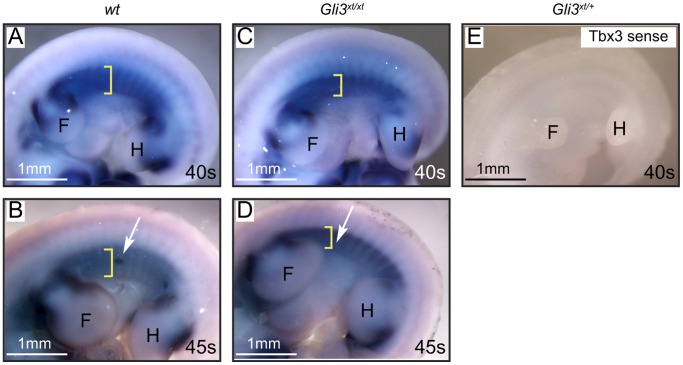
*Tbx3* mRNA expression in *Gli3^xt/xt^* embryos. Whole-mount *in situ* hybridization for *Tbx3* in wt (A, B) and *Gli3^xt/xt^* (C, D) embryos. At the 40-somite stage, wt embryos (A) express *Tbx3* mRNA in the mesenchyme between the fore- and hind-limb. This band of expression is narrower in *Gli3^xt/xt^* embryos at this stage (yellow bracket). At the 45-somite stage *Tbx3* is reduced within the mesenchyme but is induced within the epithelium of developing mammary placodes #3 (white arrow) in wt embryos (B). *Gli3^xt/xt^* embryos (D), show a narrower band of *Tbx3* expression between the fore- and hind-limb and fail to concentrate epithelial expression within placode #3 (white arrow). The control *in situ* hybridization for *Tbx3* sense probe is shown in (E). Abbreviations: F – fore-limb, H – hind-limb, s – somite.

Previously, we have shown that mice lacking Gli3 expression (*Gli3^xt/xt^*) or those genetically manipulated to misactivate Hh signaling (*Gli2^1ki/1ki^;Gli3^xt/+^)* lack mammary placodes #3 and #5 [51]. This demonstrated that for early embryonic mammary development Gli3^R^ repression of Hh signaling is essential. As *Gli3^xt/xt^* mutants lack expression of positive placodal regulators within the MR#3 and #5 regions [51], [54] we reasoned that Gli3^R^ must repress an intervening negative regulator. We hypothesized that Bmp4 could be a legitimate target of Gli3-mediated repression because it has been shown to antagonize the positive mammary placodal regulator *Tbx3* in the mammary gland and to be a target of Hh signaling in development of other organs [12], [55], [56]. To test this hypothesis, we examined the effect of Gli3 loss on *Bmp4* mRNA expression by *in situ* hybridization. As there were no significant differences in the distance between the fore- and hind-limb buds between the two genotypes (wt: 1.5±0.16 mm; *Gli3^xt/xt^*: 1.5±0.18 mm; student’s t test p = 0.934) we assessed changes in the zone of *Bmp4* expression in terms of distance from the axilla to the most distal point of expression as indicated by white dotted lines in Fig. 1B, E. In wild-type (wt) embryos (n = 6) (∼E10.5–E11.5) we observed an arc of *Bmp4* expression on the ventral flanks closely abutting the axilla of the 40-somite (Fig. 1A) and 45-somite stages (0.5±0.15 mm) (Fig. 1B) in accord with previous studies documenting *Bmp4* expression ventral to the mammary line [12]. Sections through these embryos showed *Bmp4* mRNA expression within the ectoderm and the mesenchyme (Fig. 1C, C’). By comparison, *Gli3^xt/xt^* embryos (n = 6) showed significant displacement of *Bmp4* mRNA expression between the developing fore- and hind-limb buds at the 40-somite stage (Fig. 1D) that became more pronounced by the 45-somite stage (0.8±0.22 mm; student’s t test p = 0.037) (Fig. 1E, F, F’) resulting in inappropriate *Bmp4* mRNA expression within the presumptive mammary placode #3 region (Fig. 1 arrow) that falls between somites 16 and 17 in wt. Cho et al have provided evidence that Bmp4 establishes the site of development of mammary placode #3 on the flank through mutual antagonism with Tbx transcription factors [7], [12]. Based on this model we proposed that if Gli3 functions upstream of the Bmp4/Tbx3 mechanism then *Gli3^xt/xt^* embryos would also show distortions in the *Tbx3* expression pattern. To determine if this was the case, we compared the *Tbx3* mRNA expression pattern in *Gli3^xt/xt^* embryos (n = 6) and wt (n = 6) siblings by *in situ* hybridization. Consistent with previous studies of wt embryos, we observed a wide zone of *Tbx3* expression between the fore- and hind-limbs at the 40-somite stage (Fig. 2A), that by the 45-somite stage, was reduced in intensity but strongly concentrated within the developing mammary placodes (0.5±0.1 mm) (Fig. 2B). In contrast, the *Tbx3* expression zone was narrower at both the 40- and 45-somite stages in *Gli3^xt/xt^* embryos (0.3±0.05 mm; student’s t test p = 0.004) (Fig. 2C, D) and failed to concentrate within the presumptive bud regions at the 45-somite stage (Fig. 2D). Taken together with observations of Bmp4, we conclude that Gli3 acts upstream of the *Bmp4/Tbx3* mechanism of mammary specification within the presumptive mammary placode #3 region.

### Gli3 is Required for MR#2 Invagination and Suppression of Surrounding Hair Follicles

**Figure 3:**
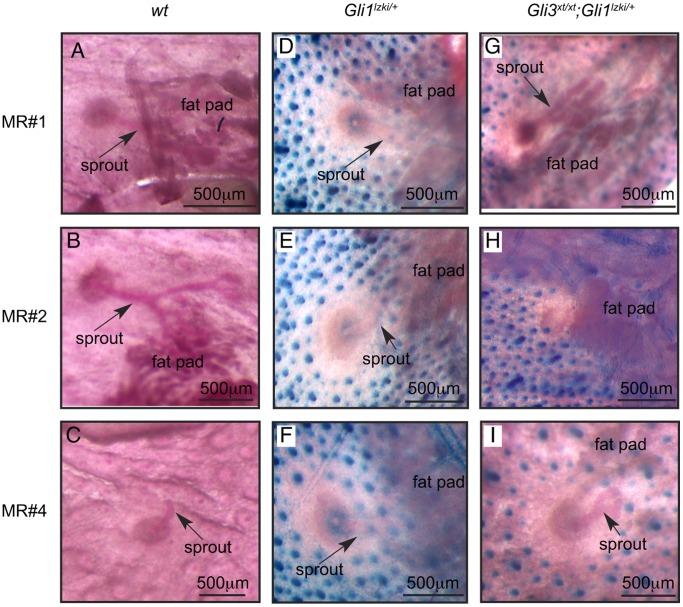
Failure of MR#2 invagination in *Gli3^xt/xt^* embryos. Analysis of the inner surface of skin whole-mounts from E18.5 wt (A, B, C) and *Gli1^lzki/+^* (D, E, F) and *Gli3^xt/xt^; Gli1^lzki/+^* (G, H, I) embryos stained with X-Gal to detect *Gli1-LacZ* reporter expression (blue) within hair follicles and counterstained with carmine alum (pink). MRs#1 (A, D, G) and #4 (C, F, I) show comparable development at E18.5 in all three genotypes: sprouts are clearly visible (arrow). In control wt (B) and *Gli1^lzki/+^* (E) embryos sprout #2 has elongated and branched several times but in *Gli3^xt/xt^; Gli1^lzki/+^* embryos (H), MR#2 shows no evidence of sprouting towards the fatpad.

**Figure 4:**
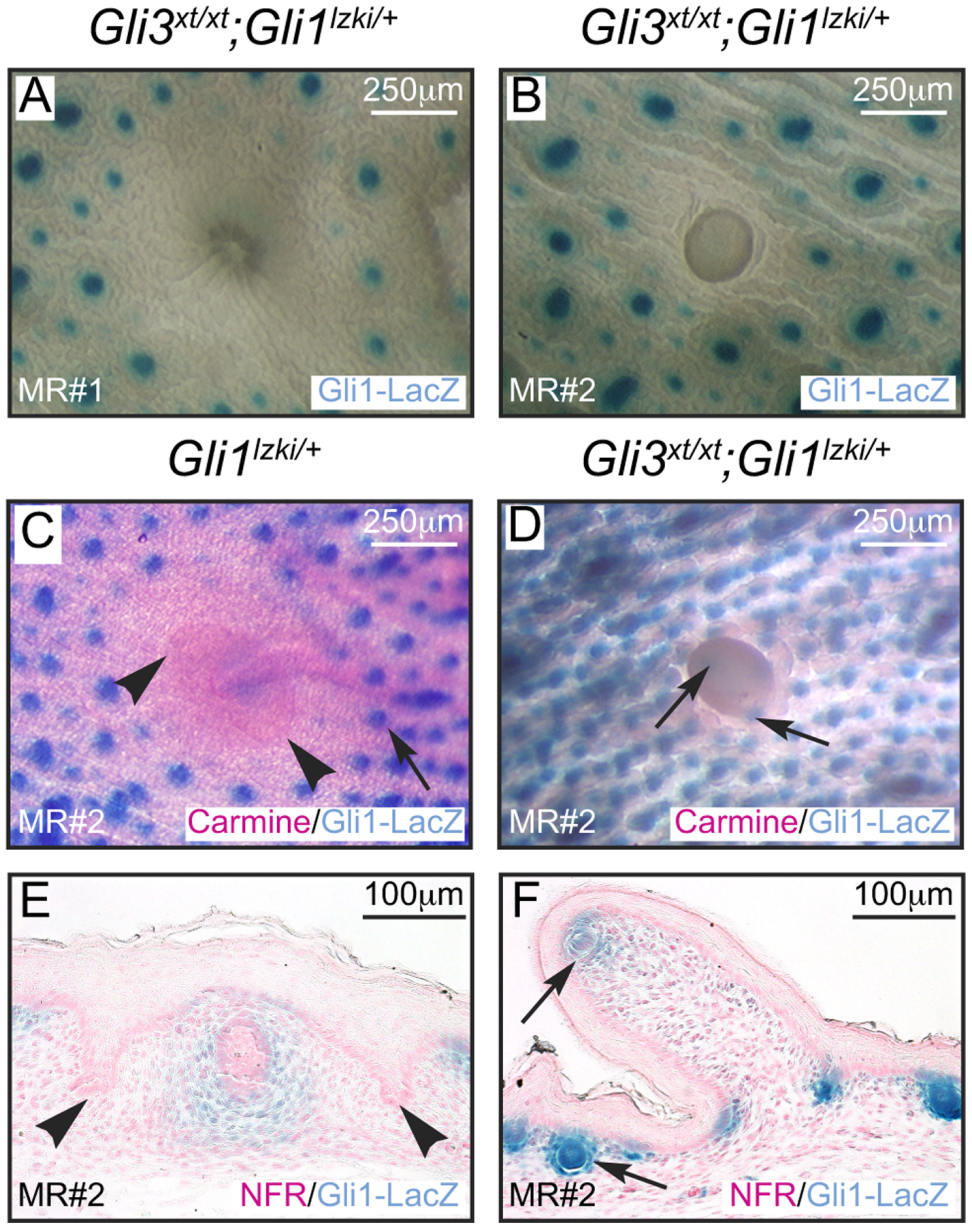
Evagination of MR#2 and encroachment of hair follicles in *Gli3* ^*xt*/xt^ embryos. Analysis of outer surface of E18.5 skin whole-mounts (A–D) stained with carmine (C, D) and sections stained with nuclear fast red (E, F) and X-Gal (blue) to detect hair follicles expressing the Gli1-LacZ reporter (A–F). MR#1 (A) from *Gli3^xt/xt^; Gli1^lzki/+^* mutants and MR#2 from control *Gli1^lzki/+^* (C) embryos show normal invagination and appropriate exclusion of hair follicles. In contrast MR#2 from *Gli3^xt/xt^; Gli1^lzki/+^* mutants (B, D) showed prominent evagination and encroachment of hair follicles.

Although placodes #3 and #5 fail to develop in *Gli3^xt/xt^* embryos (lacking Gli3), mammary placodes #1, #2 and #4 are clearly visible on the surface of E14.5 embryos, albeit with a consistent delay in #4 [51], [54]. To determine if Gli3 is required during later development we examined these remaining MRs in skin whole-mounts from E17.5 and E18.5 female *Gli3^xt/xt^* embryos (n = 49). We also took advantage of the fact that hair follicles can be distinguished from MRs in control *Gli1^lzki/+^* and in mutant *Gli3^xt/xt^*; *Gli1^lzki/+^* embryos by their engagement in Hh signaling and consequent expression of the Gli1-LacZ reporter [51]. Inspection of the inner side of the skin of control wildtype (wt) (Fig. 3A–C) and *Gli1^lzki/+^* (Fig. 3D–F) mice showed that 100% of MR#1, #2 and #4 had sprouted, with #1 and #2 penetrating the underlying dense fat pad and branching to form small ductal trees. In *Gli3^xt/xt^* mutant embryos, although MR#1 developed normally (Fig. 3G) MR#2 failed to sprout and arrested prior to invagination in 84% of cases (Fig. 3H) (Table 1). MR#4 was affected in a minority of cases (Fig. 3I) (Table 1). Examination of the outside of skin whole-mounts and histological sections confirmed that mutant MR#1 invaginated normally (Fig. 4A). However mutant MR#2 evaginated as a prominent bulge projecting from the epidermal surface (57%) (Fig. 4B and D) or was lost altogether (27%) (Table 1).

**Table 1:** Percentages of MRs showing phenotypic abnormalities in female *Gli3^xt/xt^* embryos at E18.5.

MR#	Phenotype	*Gli3^xt/xt^*	*Gli3^xt/+^*	*Gli3^+/+^*
		(n = 49)	(n = 90)	(n = 58)
**1**	Loss	4	0	0
**2**	Evagination	57	0	0
	Loss	27	0	0
**4**	Evagination	6	0	0
	Imp. Invag.	4	0	0
	Loss	4	0	0

Numbers represent percentages of MRs showing loss, evagination or impairment in invagination from a total ‘n’. Abbreviations: Imp. Invag: Impaired Invagination; MR: mammary rudiment; xt: extra toe mutation; n: total number of MRs analyzed.

Suppression of hair follicle formation within the designated nipple sheath is an important aspect of late embryonic mammary development [11]. To determine if this process was affected by loss of Gli3 activity, we analyzed hair follicle suppression in control *Gli1^lzki/+^* and mutant *Gli3^xt/xt^*; *Gli1^lzki/+^* embryos. Hair follicles were appropriately excluded from the presumptive nipple areolar zone of all MRs from control *Gli1^lzki/+^* embryos (Fig. 3D–F and Fig. 4C) as well as from MR#1 and MR#4 of mutant *Gli3^xt/xt^*; *Gli1^lzki/+^* embryos (Fig. 3G and I; Fig. 4A). In contrast, in mutant *Gli3^xt/xt^*; *Gli1^lzki/+^* embryos, ectopic hair follicles expressing Gli1-LacZ were observed inappropriately close to the base (Fig. 4D) and even at the tip of the evaginated MR#2 (Fig. 4F).

### Loss of Gli3 Impairs MR#2 Mammary Mesenchyme Specification

**Figure 5:**
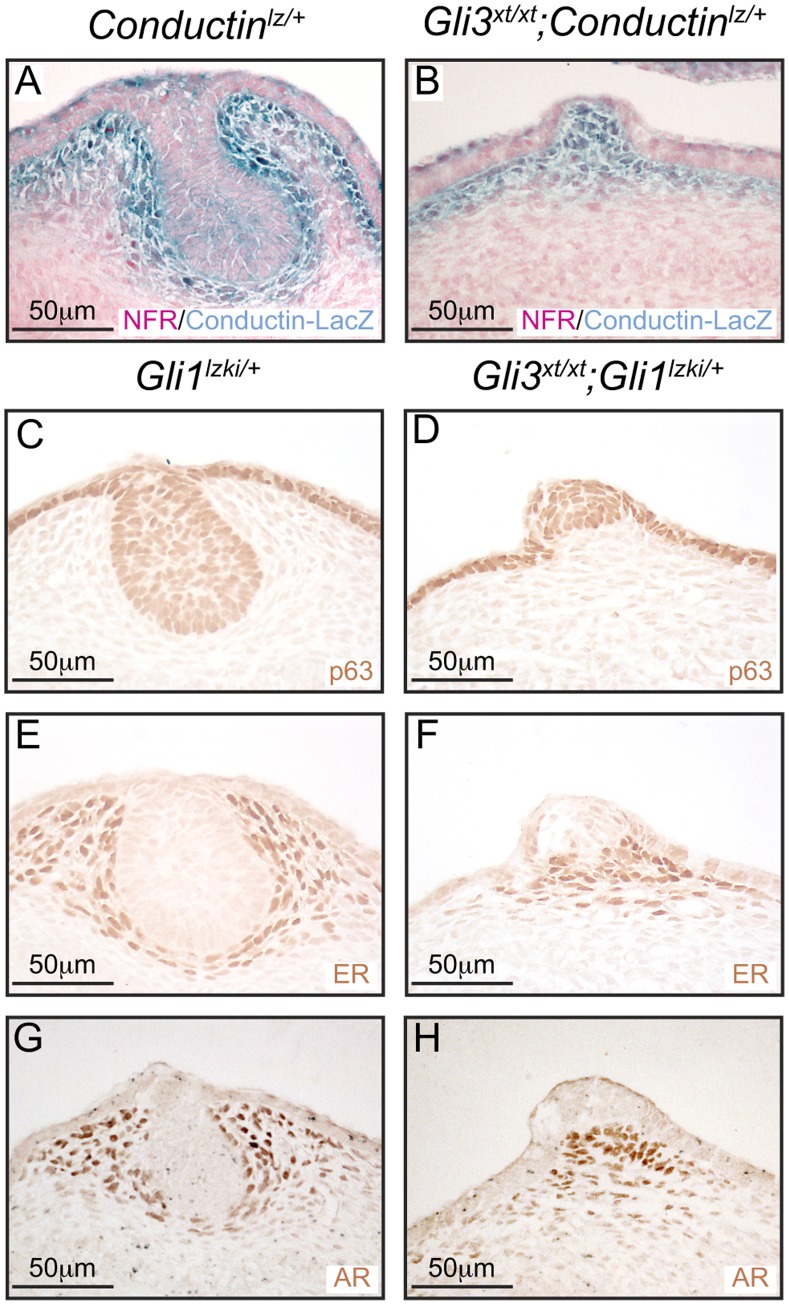
Mammary mesenchyme specification in E14.5 *Gli3^xt/xt^* embryos. (A, B) Analysis of sections of MR#2 stained with X-Gal (blue) for expression of Conductin-LacZ reporter and counterstained with NFR. Control *Conductin^lz/+^* embryos (A) show Wnt/β-catenin signaling pathway activity in the mammary mesenchyme and central epithelial cells whereas *Gli3^xt/xt^; Conductin^lz/+^* embryos (B) show activation only within the mesenchymal compartment. Immunohistochemical analysis of serial sections from control E13 *Gli1^lzki/+^* (C, E, G) and mutant *Gli3^xt/xt^;Gli1^lzki/+^* (D, F, H) embryos for expression of (C, D) p63, (E, F) ER and (G, H)AR. Note the epithelium of *Gli3^xt/xt^;Gli1^lzki/+^* mutant embryos fails to invaginate (D), the mammary mesenchyme shows no histological evidence of condensation and few cells express ER (F) and AR (H).

**Figure 6:**
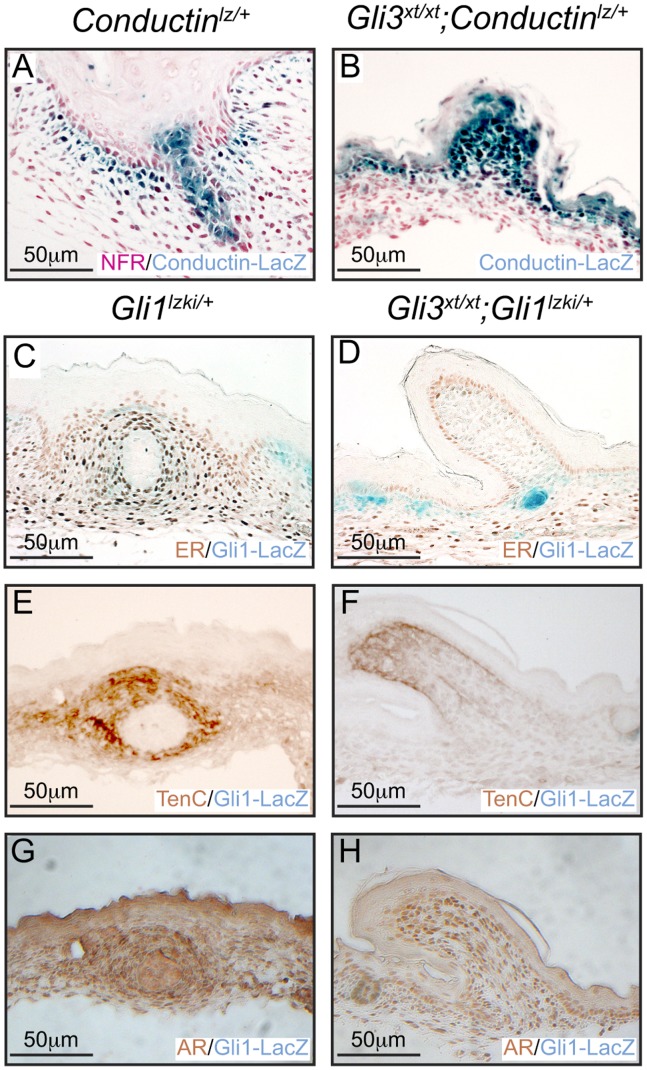
Gain of epithelial Wnt signaling and maintenance of mammary mesenchyme markers is compromised in MR#2 of *Gli3^xt/xt^* embryos. Analysis of X-Gal and NFR stained sections from MR#2 at E18.5. (A) Conductin-LacZ is robustly expressed within the epithelial mammary sprout of control *Conductin^lz/+^* embryos whereas (B) mutant *Gli3^xt/xt^; Conductin^lz/+^* embryos lack expression within the epithelium and maintain robust mesenchymal expression. (C–J) Analysis of serial sections for mammary mesenchyme markers by immunohistochemistry revealed that ER (C), Tenascin C (E) and AR (G) are maintained in control *Gli1^lzki/+^* embryos. In contrast, ER was lost (D) and Tenascin C and AR expression were weakened (F, H) in mutant *Gli3^xtxt^;Gli1^lzki/+^* embryos.

A series of elegant experiments has demonstrated that complex reciprocal epithelial-mesenchymal signaling regulates MR invagination and suppression of surrounding hair follicles [3]. To investigate the status of the mammary mesenchyme specification we first investigated Wnt/β-catenin signaling pathway activity by crossing *Gli3^xt/+^* to *Conductin^lz/+^* heterozygous LacZ knock-in reporter lines. *Conductin* is expressed constitutively in response to canonical Wnt/β-catenin signaling and its product negatively regulates the pathway [57], [58]. In control *Conductin^lz/+^* mice the Conductin-LacZ reporter was expressed in the mammary mesenchyme and within central epithelial cells of MR#2 (Fig. 5A) whereas in the evaginated MR#2 of *Gli3^xt/xt^;Conductin^lz/+^* embryos Conductin-LacZ expression was present only within the mesenchymal compartment (Fig. 5B). Next we examined serial sections of *Gli3^xt/xt^*;*Gli1^lzki/+^* E13 embryos stained estrogen receptor (ER), and androgen receptor (AR) antibodies as markers of mammary mesenchyme specification and with p63 antibodies to detect the epithelial layer. In control *Gli1^lz/+^* embryos MR#2 comprised a compact p63-positive epithelial bulb beneath the epidermis (Fig. 5C), surrounded by a condensed ring of ER-positive and AR-positive mammary mesenchymal cells (Fig. 5E and G respectively). In *Gli3^xt/xt^*;*Gli1^lzki/+^* embryos, MR#2 comprised a raised epithelial placode of p63-positive cells (Fig. 5D). However few underlying mesenchymal cells expressed ER and AR (Fig. 5F and H respectively), and these failed to condense. By E18.5, Conductin-LacZ reporter expression had switched in control *Conductin^lz/+^* embryos: being diminished within the mesenchyme and robustly upregulated within the mammary sprout (Fig. 6A). In contrast, *Gli3^xt/xt^*;*Conductin^lz/+^* embryos maintained robust reporter expression in the MR#2 mammary mesenchyme but failed to upregulate expression within the epithelium (Fig. 6B). Control *Gli1^lz/+^* embryos continued to show robust expression of ER, AR and Tenascin C within the mammary mesenchyme surrounding the MR#2 sprout (Fig. 6 C, E, G). However, *Gli3^xt/xt^*;*Gli1^lz/+^* embryos had lost mesenchymal ER expression (Fig. 6D) and showed weak Tenascin C and AR expression (Fig. 6F and H) in evaginated MR#2 and lacked all histological signs of mammary mesenchyme condensation (Fig. 6C–H). Collectively these results show that, although mammary mesenchymal specification is initiated normally, maintenance of mammary mesenchymal markers and gain of epithelial Wnt signaling is compromised in MR#2 of *Gli3^xt/xt^* embryos.

### Gli3 is Required for Sexual Dimorphism during Mammary Development

**Figure 7:**
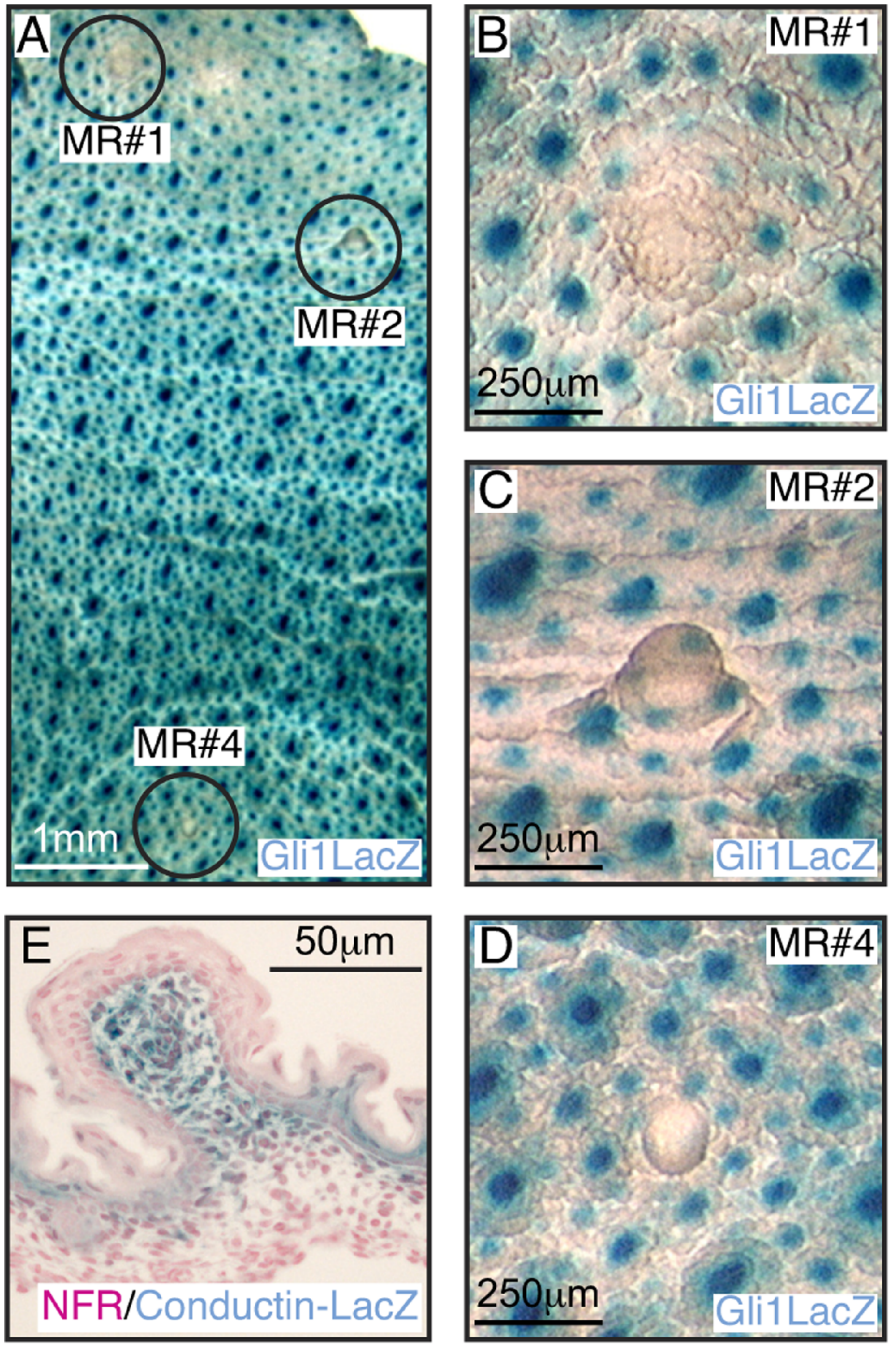
Sexual dimorphism is lost in *Gli3^xt/xt^* embryos. X-Gal stained skin whole-mounts from E18.5 male *Gli3^xt/xt^;Gli1^lzki/+^* embryos show retention of MR#1, #2 and #4 (A). Examination of skins at high power revealed that MR#1 does not protruded from the surface of the skin (B), whereas MR#2 and #4 clearly evaginate (C, D). Elevated Wnt signaling activity can be seen in mesenchymal cells of protruding MR#2 from *Gli3^xt/xt^;Conductin^lz/+^* embryos at this stage (E).

**Figure 8:**
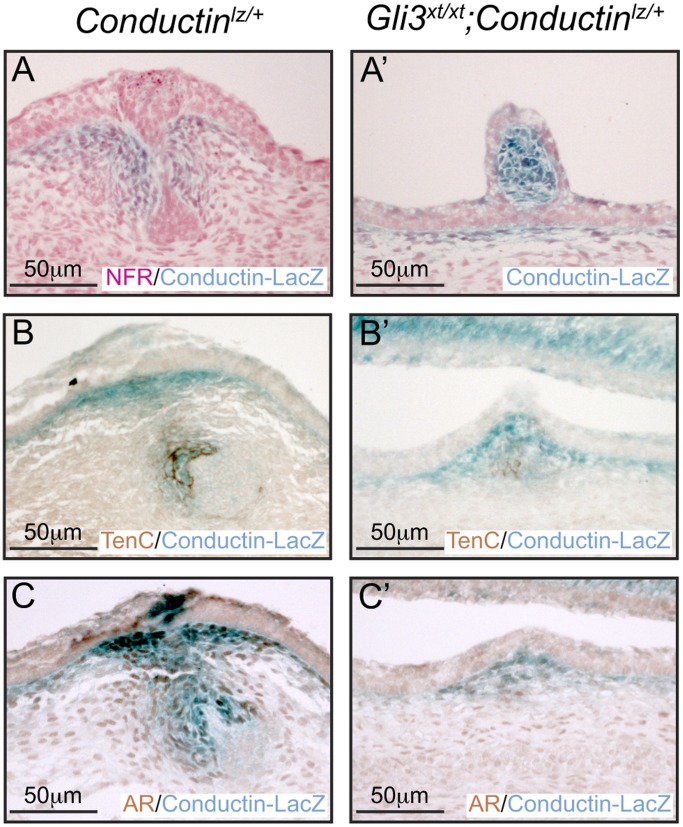
Mammary mesenchyme specification is impaired in male *Gli3^xt/xt^* embryos. X-Gal stained sections from control *Conductin^lz/+^* embryos showed appropriate constriction of mesenchymal cells coincident with the expression of Conductin-LacZ reporter (A), Tenascin C (B) and AR (C). However, mesenchymal cells surrounding the protruding MR#2 of *Gli3^xt/xt^;Conductin^lz/+^* embryos retained mesenchymal Conductin-LacZ expression (A’) and showed weak Tenascin C (B’) and AR (C’) expression.

Next we asked whether the observed impairment in the mammary mesenchymal specification program has functional consequences for MR formation in males. Around E13 a surge of secreted androgens in male embryos induces mammary mesenchymal cells to encapsulate and cause the mammary bulb to atrophy [3], [9], [15], [17], [18], [19], [20], [59]. To determine whether Gli3 activity influences this process we looked for evidence of inappropriate retention of MRs in E14.5 male embryos. In control *Gli3^+/+^* embryos all MRs were appropriately lost by E16.5 (Table 2). Male *Gli3^xt/xt^* embryos, like their female counterparts failed to form MR#3 and MR#5. However, in *Gli3^xt/xt^* males 66% of MR#1 were retained at E16.5 but only 15% by E18.5 suggesting that their normal atrophy occurred but was delayed (Table 2). However, MR#2 and MR#4 showed very high rates of retention at both E16.5 and E18.5 (Table 2). The majority of these retained MRs#2 and #4 were evaginated (Table 3; Fig. 7C, D). In control E14.5 *Gli3^+/+^* male embryos MRs showed robust expression of Conductin-LacZ, Tenascin C and AR in the zone of mesenchymal constriction around the epithelial bulb (Fig. 8A–C). Mutant *Gli3^xt/xt^;Conductin^lz/+^* embryos showed mesenchymal Conductin-LacZ expression in the three remaining MRs but epithelial Wnt signaling was absent (Fig. 7E and Fig. 8), Tenascin C and AR were expressed in very few cells and the MRs were frequently evaginated (Fig. 8A’–C’, Table 3). Thus Gli3 activity exerts a significant influence on sexual dimorphism.

**Table 2:** Percentages of MRs showing retention in male *Gli3^xt/xt^* embryos at E16.5 and E18.5.

	Retention (E16.5)			Retention (E18.5)		
MR#	*Gli3^xt/xt^*	*Gli3^xt/+^*	*Gli3^+/+^*	*Gli3^xt/xt^*	*Gli3^xt/+^*	*Gli3^+/+^*
	(n = 30)	(n = 58)	(n = 18)	(n = 54)	(n = 88)	(n = 56)
**1**	66	0	0	15	0	0
**2**	77	0	0	69	0	0
**4**	78	0	0	61	0	0

Numbers represent percentages of MRs retained on male skin whole-mounts from a total ‘n’. Abbreviations: E: embryonic day; n: total number of putative sites for MRs on male skin whole mounts.

**Table 3:** Percentages of evaginated MRs in male *Gli3^xt/xt^* embryos at E16.5 and E18.5.

	Evagination (E16.5)			Evagination (E18.5)		
MR#	*Gli3^xt/xt^*	*Gli3^xt/+^*	*Gli3^+/+^*	*Gli3^xt/xt^*	*Gli3^xt/+^*	*Gli3^+/+^*
	(n = 30)	(n = 58)	(n = 18)	(n = 54)	(n = 88)	(n = 56)
**1**	7	0	0	2	0	0
**2**	60	0	0	63	0	0
**4**	56	0	0	61	0	0

Numbers represent percentages of MRs that protrude from the surface of male skin whole-mounts from a total ‘n’. Abbreviations: E: embryonic day; n: total number of putative sites for MRs on male skin whole mounts.

### Gli3 Acts as a Repressor of Hh Signaling during Late Mammary Development

**Figure 9:**
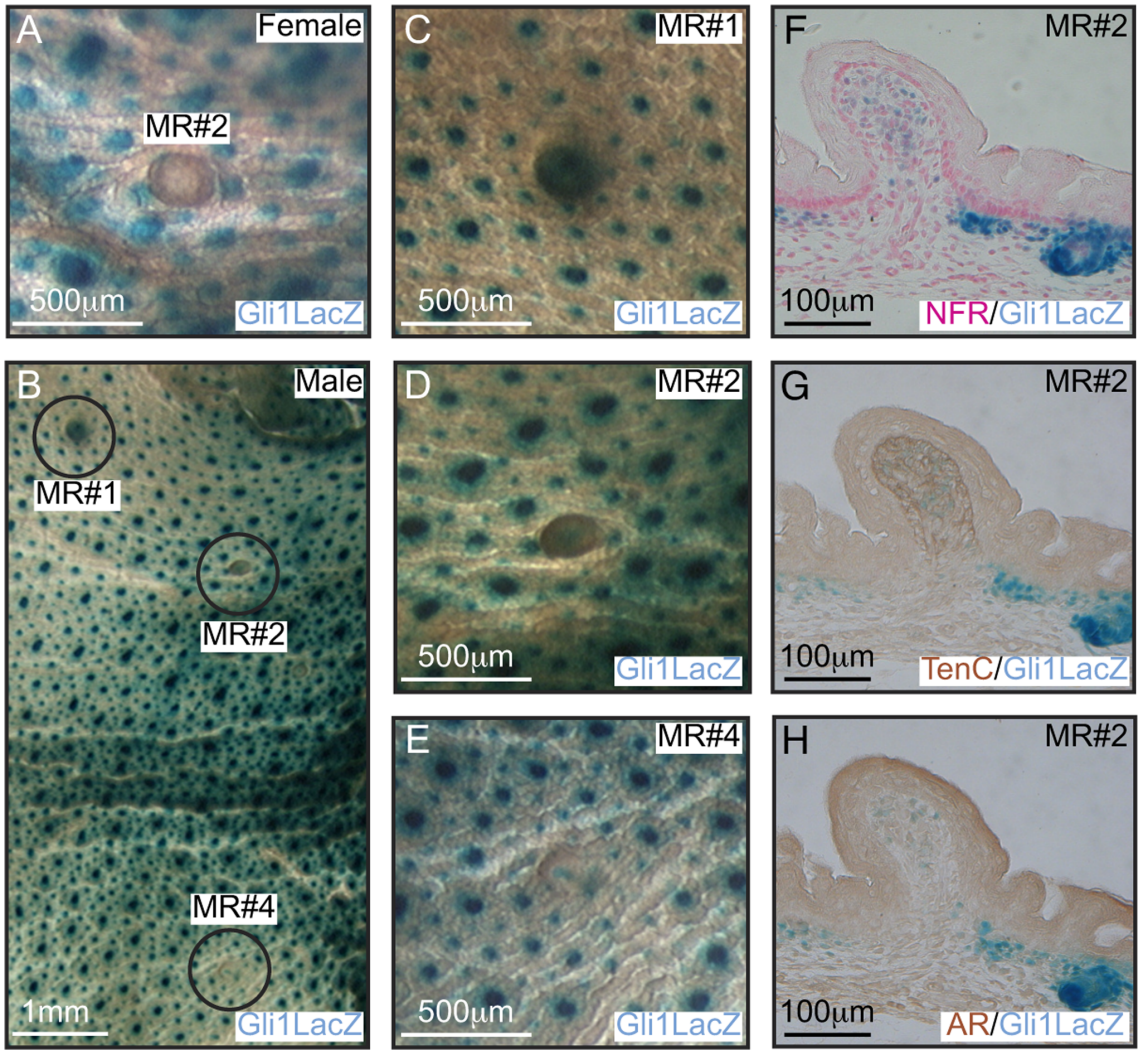
Misactivation of Hh signaling detrimentally affects MR invagination and hair follicle suppression in females and eradication of MRs in males. X-Gal stained whole-mounts (A–E) and sections (F-H) of *Gli2^1ki/1ki^;Gli3^xt/+^;Gli1^lzki/+^* embryos were examined at E18.5. In whole-mounts of female skins, MR#2 protruded prominently and showed encroachment of hair follicles inappropriately close to the evaginated MR (A). Examination of male skin whole-mounts revealed retention of MR#1, #2 and #4 at low (B) and high power (C, D, E respectively), similar to that seen in *Gli3^xt/xt^;Gli1^lzki/+^* embryos. Serial sections through a male MR#2 from *Gli2^1ki/1ki^;Gli3^xt/+^;Gli1^lzki/+^* embryos showed Gli1-LacZ-positive hair follicles close to the protruding bud (F; NFR counterstain) and weak expression of Tenascin C (G) and loss of AR (H) by immunohistochemistry.

Our results show that lack of Gli3 expression severely compromises MR#2 invagination in both sexes and leads to inappropriate retention of MRs#1, #2 and #4 in males. To test if Gli3 functions as an activator or repressor of Hh signaling during these later stages of mammary development we crossed *Gli2^1ki/+^* mice, which drives the expression of constitutively active Gli1 transactivator under the control of Gli2 promoter to *Gli2^1ki/+^*; *Gli3^xt/+^;Gli1^lzki/lzki^* mice. The genotypes arising from this cross alter the Gli^R^:Gli^A^ ratio to progressively misactivate the pathway [36], [51]. Analysis of skin whole-mounts from E18.5 females showed that misactivation of the Hh pathway in *Gli2^1ki/1ki^*;*Gli3^xt/+^;Gli1^lzki/+^* embryos (n = 10) produces the same spectrum of phenotypes seen in *Gli3^xt/xt^*;*Gli1^lzki/+^* embryos: MR#2 was prominently evaginated (60% (Fig. 9A) and MRs#1 and #4 showed mild impairment of invagination in 20% of cases (Table 4). Hair follicles, demarcated by expression of the Gli1-LacZ reporter, inappropriately encroached around the protruding MR#2 of *Gli2^1ki/1ki^*;*Gli3^xt/+^;Gli1^lzki/+^* embryos (Fig. 9A), in a manner similar to that shown previously for *Gli3^xt/xt^*;*Gli1^lzki/+^* embryos (Fig. 4). Similarly, in male embryos, Hh pathway misactivation leads to significant rates of retention of MR#1, MR#2 and MR#4 (Fig. 9B–E; Table 5), evagination of MR#2 and reduced expression of Tenascin C and AR (Fig. 9F–H).

**Table 4:** Percentages of MRs showing phenotypic abnormalities in female *Gli2^1ki/1ki^; Gli3^xt/+^* embryos at E18.5.

MR#	Phenotype	*Gli2^1ki/1ki^; Gli3^xt/+^*	*Gli2^1ki/1ki^*	*Gli2^1ki/+^; Gli3^xt/+^*	*Gli2^1ki/+^*	*Gli3^xt/+^*	*Gli3^+/+^*
		(n = 10)	(n = 14)	(n = 8)	(n = 8)	(n = 8)	(n = 10)
1	Imp. Invag.	20	0	0	0	0	0
2	Evagination	60	0	0	0	0	0
4	Evagination	10	0	0	0	0	0
	Imp. Invag.	10	0	0	0	0	0
	Loss	0	0	13	0	0	0

Numbers represent percentages of MRs showing loss, evagination or impairment in invagination from a total ‘n’. Abbreviations: Imp. Invag: Impaired Invagination; n: total number of MRs analyzed.

**Table 5:** Percentages of MRs showing retention and evagination in male *Gli2^1ki/1ki^; Gli3^xt/+^* embryos at E18.5.

MR#	Phenotype	*Gli2^1ki/1ki^; Gli3^xt/+^*	*Gli2^1ki/1ki^*	*Gli2^1ki/+^; Gli3^xt/+^*	*Gli2^1ki/+^*	*Gli3^xt/+^*	*Gli3^+/+^*
		(n = 32)	(n = 10)	(n = 8)	(n = 14)	(n = 16)	(n = 16)
1	Retention	69	25	0	0	0	0
	Evagination	0	0	–	–	–	–
2	Retention	44	0	25	0	0	0
	Evagination	44	–	0	–	–	–
4	Retention	16	0	13	0	0	0
	Evagination	9	–	0	–	–	–

Numbers represent percentages of MRs that are retained or protrude [in brackets] from the surface of male skin whole-mounts from a total ‘n’. Abbreviations: n: total number of putative sites for MRs on male skin whole mounts.

### The Hh Pathway is Activated in Developing and Adult Nipple

**Figure 10:**
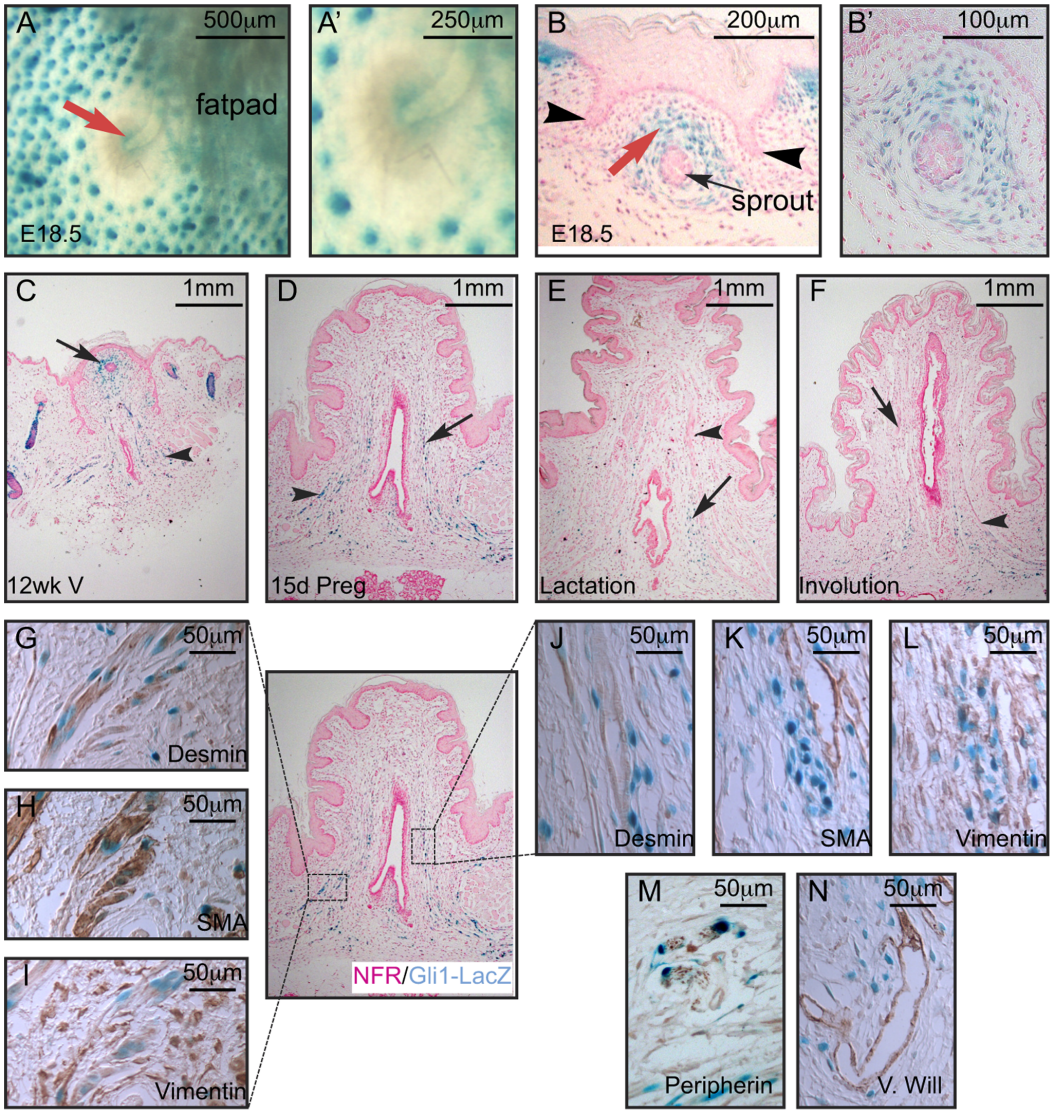
Expression and modulation of *Gli1-LacZ* in the adult nipple during the pregnancy cycle. Gli1-LacZ expression is visible at the neck of the mammary sprout (red arrow) in skin whole-mounts of E18.5 *Gli1^lzki/+^* embryos at low (A) and higher power (A’). Histological section through the sprout shows expression of Gli1-LacZ within the stroma (red arrow) surrounding the sprout (black arrow) underneath the nipple sheath (black arrowheads) at low (B) and higher power (B’). *Gli1-lacZ* is expressed within the dermal component but not the epithelium in virgin (C), mid pregnant (D), lactating (E) and involuting (F) nipples. Immunohistochemistry for desmin (G, J), SMA (H, K) and vimentin (I, L) on serial sections of a 15.5 day pregnant nipple demonstrated that *Gli1-lacZ* was expressed in both smooth muscle cells and fibroblasts but not myofibroblasts. *Gli1-lacZ* was also found near and surrounding peripherin positive nerve tracts (M) and both Von Willebrand positive vessels (N).

In contrast to the requirement for repression of Hh signaling in mammary rudiment development, we found that positive Hh signaling occurs within the developing nipple. A small ring of Gli1-LacZ expression was observed at E18.5 in *Gli1^lzki/+^* embryonic skin whole-mounts (Fig. 10A) and histological sections (Fig. 10B) within the mesenchyme around the neck of the lactiferous duct and under the nipple sheath. Postnatally, the mammary mesenchyme develops into highly specialized nipple mesenchyme (Fig. 10C–F). Immunohistochemical analysis of nipple sections defined a number of cell types undergoing Hh signaling in the postnatal nipple. Des+;SMA+;Vim- smooth muscle cells, showed Gli1-LacZ expression during puberty, pregnancy and involution but lost reporter expression during lactation (Fig. 6G, H, I). Gli1-LacZ-positive Des-;SMA-;Vim+ fibroblasts surrounded the lactiferous duct (Fig. 10 J, K, L). Minor subsets of Vim+ Gli1-LacZ-positive fibroblasts were found encasing peripherin-positive nerve tracts (Fig. 6M) and in close association with Von-Willebrand factor positive vessels (Fig. 6N).

## Discussion

The main findings of our study are threefold. First, that Gli3^R^ lies upstream of Bmp4/Tbx3 specification of placode #3. Second, that at later stages Gli3^R^ significantly influences the maintenance of mammary mesenchyme specification and function. Third, that Gli3 impinges on these developmental events via repression of Hh signaling. In contrast we document that positive Hh signaling occurs during embryonic and postnatal nipple development.

### Gli3^R^ Acts Upstream of Bmp4/Tbx3 Patterning

Our data show that loss of Gli3 results in inappropriate expansion of *Bmp4* and failure of *Tbx3* to concentrate within the presumptive MR#3 region. The positive role of *Tbx3* in MR development is well documented [7], [60], [61]. Firstly, Tbx3 is expressed within mammary placodes at E11.75 and *Tbx3* ^−/−^ mice lack most mammary buds [7], [12]. During postnatal mammary development, haploinsufficient *Tbx3^+/−^* mice display significantly underdeveloped ductal trees at puberty and conversely, inducible Tbx3 overexpression accelerates mammary epithelial cell proliferation resulting in mammary hyperplasia [7], [62]. In humans, heterozygous mutations in *TBX3* result in Ulnar-Mammary Syndrome, which is characterized by mammary hypoplasia [63]. Aberrations in human *Tbx3* gene have also been implicated in breast cancer [64], [65], [66], [67], [68]. The fact that loss of Gli3 repression results in loss of upregulation at the site of placodal #3 points to the involvement of an intervening *Tbx3* suppressor, and previous studies suggest that Bmp4 is the most likely candidate for this role [12]. *Tbx3* is a direct transcriptional target of Bmp/Smad activity during the development of limb buds, retina and adult brain [69], [70], [71]. Antagonism between *Bmp4* and *Tbx3* has been shown to be critical for formation of the mammary line within the presumptive MR#3 region [12]. Previous studies have placed *Gli3* upstream of the positive placodal regulator *Fgf10* [54]. However we have shown that gain of Hh signaling negatively regulates placodes #3 formation [51]. Hence Gli3 must repress an intervening Hh-dependent placodal repressor. The finding that loss of Gli3^R^ expands the zone of *Bmp4*, a known antagonist of the positive placodal regulator Tbx3, suggests that Bmp4 may be this intervening repressor. Consistent with this concept, connections between Hh signaling and mesenchymal *Bmp4* expression have been documented during development of other tissues such as the hindgut, kidney and prostate [55], [56], [72], [73], [74]. Supporting the possibility that *Bmp4* is a direct transcriptional target of positive Hh signaling and Gli3^R^ repression, Gli binding sites are present in the murine *Bmp4* promoter and transfection of cDNAs encoding Gli1 or Gli3^A^ has been shown to activate a human *BMP4* promoter-reporter construct in COS-7 cells [75], [76]. Collectively these findings suggest a model in which *Gli3* acts upstream of *Fgf10* and also upstream of *Bmp4/Tbx3* in the latter case by acting to repress Hh-activation of Bmp4 thereby relieving antagonism on the positive placodal regulator Tbx3.

### Gli3^R^ Influences Mammary Mesenchyme Specification and Function

Our results show that although early Gli3^R^ function is essential for the formation of placodes #3 and #5, it is not required for the early development of placodes #1, #2 and #4 [51], [54]. It has been well documented that mammary placodes form in a specific temporal order (#3, #4, #1, #5, #2) and that each pair has a unique set of regulatory requirements [1], [77]. In *Gli3^xt/xt^* mutants placodes #1 and the majority of #4 go on to sprout and branch despite loss of pathway repression [51]. However, loss of Gli3 activity in *Gli3^xt/xt^* mutants produces profound effects on the later development of MR#2, which forms a large evaginated protrusion. A previous report has suggested that higher proliferation of mesenchymal cells and inability of the adjacent ectoderm to undergo apoptosis contributes to this protrusion and that MR#2 nevertheless sprouts [4]. In contrast, our results show that 84% of MR#2 fail to invaginate or sprout and remain evaginated or are lost altogether. A series of elegant experiments has shown that invagination and sprout downgrowth are regulated by reciprocal epithelial-mesenchymal signaling that lead to specialization and subsequent condensation of mammary mesenchyme together with suppression of surrounding hair follicles. These studies showed: (1) PTHrP released from the bud specifies the surrounding condensed mammary mesenchyme inducing expression of reporters of β-catenin signaling and a suite of mesenchymal markers including Lef1, hormone receptors and Tenascin C [9], [11], [13], [14], [16], [59], [77], [78], [79]; (2) Lef-independent Wnt signaling is first required in the mesenchyme for mammary mesenchyme specification but Wnt signaling occurs in both compartments and Lef-dependent activity is required later for sprouting [5], [59], [80], [81]; (3) In females PTHrP-dependent upregulation of mesenchymal *BmpR1a* expression also increases mesenchymal *Msx2* expression, which inhibits hair follicle cell fate within the overlying nipple epidermis [11], [13], [14], [16], [82]; (4) In males androgen stimulation of AR expression leads to their detachment and loss of this PTHrP-dependent AR expression in PTHrP^−/−^ mice manifests as loss of sexual dimorphism [15], [17], [18], [19], [20], [59]. Our results show that *Gli3^xt/xt^* mutants initiate mammary mesenchyme specification, evidenced by mesenchymal expression of Conductin-LacZ Wnt reporter, but fail to maintain mammary mesenchymal ER expression or to upregulate epithelial Wnt signaling. The loss of these markers suggests that the feedback signaling loop between the mammary mesenchyme and overlying epithelial compartments is defective. This likely accounts for the failure to establish nipple identity during subsequent development and the reversion of the overlying epithelium to an epidermal fate evidenced by aberrant formation of ectopic hair follicles within the epidermis of the evaginated bud. The functional significance of this mammary mesenchyme impairment is also reflected by the loss of sexual dimorphism. Despite the low levels of expression of AR and Tenascin C clearly designating the sites for MR formation in both sexes, the failure to maintain robust expression of these markers likely impairs the androgenic response that would normally induce their demise in males leading to the aberrant retention of primitive and frequently evaginated MRs#1, #2 and #4 in *Gli3^xt/xt^* male embryos.

### Gli3 Acts via Repression of Hh Signaling during Late Embryonic Mammary Development

Gli3 proteins occur within mammalian cells either in a Hh-depdendent full-length transcriptional-activator capacity (Gli3^A^) or in the absence of Hh signals, are proteolytically cleaved into truncated repressor proteins (Gli3^R^). Highly cell-contextual and opposing functions of Gli3 have been documented in different mammalian systems: For example, in spinal cord, skeletal muscle and stomach Gli3’s primary function is that of Hh-activation whereas Gli3^R^ is the critical repressor of Hh signaling pathway in hair, teeth, limb and lung development [35], [43], [44], [45], [83], [84], [85], [86], [87]. A critical balance of Gli3^R^:Gli3^A^ ratio is maintained within mammalian cells for proper execution of Hh signaling pathway (reviewed in [88]). Our results show that deliberate Hh-pathway misactivation (*Gli2^1ki/1ki^;Gli3^xt/+^*) produces the same spectrum of phenotypic aberrations as loss of Gli3 in both sexes: MR#2 fails to invaginate, to upregulate epithelial Wnt reporter expression, to appropriately condense mammary mesenchyme and to suppress surrounding hair follicles. In addition, expression of mesenchymal markers AR and Tenascin C is more prominently impaired in misactivated MRs. These results provide genetic evidence that Gli3 acts as a repressor of Hh signaling and not in some Hh-independent or Hh transactivator capacity. We conclude that Gli3 functions as a repressor of Hh signaling and significantly influences three events in mammary development: MR invagination, hair follicle suppression and eradication of MRs in males.

### Positive Hh Signaling in Embryonic Nipple Development

Although this study shows that repression of the Hh pathway is essential for both early and late embryonic mammary development, we found evidence that the Hh pathway becomes activated during embryonic nipple development. Gli1-LacZ expression occurs ∼E17.5 within the mammary mesenchyme underlying the developing nipple sheath and surrounding the lactiferous ducts of all wild-type glands. Nipples are sites of regional epidermal specialization and their formation is dependent upon inductive signals from the underlying ventral dermis to the overlying ventral epidermis [11], [13], [14], [78], [89]. Due to the poor survival of *Gli2^lzki/lzki^* embryos at this stage we have been unable to address whether positive Hh signaling is essential for embryonic nipple development [36], [90]. However the timing of the Gli1-LacZ reporter expression suggest the possibility that positive Hh signaling may participate in this patterning process. Positive Hh signaling continues postnatally within the nipple connective tissue and is robust in cell-types that distinguish nipple stroma from adjacent ventral dermis. For example, Gli1-LacZ is expressed within cells running circumferential to the lactiferous duct that provide mechanical support to the nipple during suckling. Hh signaling is also active in smooth muscle of the nipple sphincter that play important functions during the milk let down response. Gli1-LacZ is also expressed in cells surrounding capillaries, which are abundant in nipple and serve to nourish the thickened epidermis, and surrounding nerve tracts that send stimuli leading to oxytocin release [14], [82], [89]. These histological specializations of the nipple connective tissue form during the first few weeks after birth but are predetermined during embryonic exposure of the mammary mesenchyme to PTHrP, as demonstrated by their *de novo* induction in the entire ventral dermis of female mice overexpressing PTHrP under the control of the keratin 14 promoter [82], [91]. The relationship between Hh activity and the development of the nipple stroma remains an important question for future study.

## Experimental Procedures

### Mice

The following mice were maintained on an outbred background. *Gli1^lzki/+^*, *Gli2^1ki/+^*, *Gli3^xt/+^* mice were generously provided by Dr. Alexandra Joyner, Memorial Sloan Kettering Cancer Institute, and constructed as described [36], [37] and *Conductin^lz^* ^/+^ (also called *Axin2^lz^* ^/+^) mice were a gift from Dr. Franke Costantini, Columbia University [57], [58]. All animal protocols were approved by the Institutional Animal Care and Use Committee (IACUC) of New York University School of Medicine (NYUSOM). The animals were monitored and cared for daily at the NYUSOM Skirball Central Animal Facility (SCAF), which were maintained to be sterile and clean, requiring full gowning (head covers, masks, gowns, gloves and booties) procedures to ensure that the animals are not exposed to outside pathogens. Before sacrifice, mice were first anaesthetized using carbon dioxide and then euthanized by cervical dislocation. All animal care and euthanasia procedures adhered to the guidelines specified by the NYUSOM Division of Laboratory Animals Resources (DLAR: www.med.nyu.edu/dlar).

### Whole-mount X-Gal Staining

For detection of LacZ expression, embryos or tissues were fixed in 4% paraformaldehyde (PFA, Sigma Aldrich, St. Louis, MO) diluted in phosphate buffered saline (PBS) for 30 minutes, followed by four 15 minutes washes in rinse buffer (2 mM MgCl_2_, 0.1% sodium deoxycholate, 0.2% NP40 prepared in PBS). X-Gal staining was carried out at room temperature for 2–3 hours in staining buffer (5 mM potassium ferricyanide, 5 mM potassium ferrocyanide, 1 mg/ml 5-bromo-4-chloro-3-indolyl-b-D-galactopyranoside (X-Gal, Denville Scientific, South Plainfield, NJ) prepared in rinse buffer). After staining, embryos and tissues were washed in PBS, post-fixed for overnight in 4% PFA at 4°C and viewed under a Zeiss Axiovert (Oberkochen, FRG) brightfield dissecting microscope.

### Whole-mount *in situ* Hybridization

Embryos were fixed overnight in 4% PFA diluted in PBS, dehydrated in methanol and stored at –20°C. Before hybridization embryos were rehydrated, bleached by incubating for 30 minutes in 6% hydrogen peroxide, treated with 4 µg/ml proteinase K for 10 minutes, washed in 2 mg/ml glycine, then fixed in 4% PFA for 20 minutes. All solutions were made up in PBS-T (PBS, 1% Tween-20) and three 5-minute PBS-T washes followed each step. Embryos were prehybridized for 2–3 hours in 50% formamide 5X SSC, 50 µg/ml tRNA, 1% SDS and 50 µg/ml heparin followed by hybridization overnight at 70°C in the same buffer containing 2 µg/ml of digoxigenin (DIG) labeled *Bmp4* or *Tbx3* probe. Following several washes, DIG was detected by overnight incubation at 4°C in alkaline phosphatase (AP) labeled anti-DIG Fab’ fragments (Roche, Indianapolis, IN). Color was developed with BM-purple AP substrate (Roche). Embryos were postfixed in 4% PFA, embedded in paraffin and sectioned. The distance between the fore- and hind- limb was relatively uniform in all genotypes therefore we determined the extent of Bmp4 expression by measuring the distance between the base of the fore-limb bud (axilla) and the most posterior tip of expression (indicated by white dotted lines in Fig. 1B, E) and compared the distances in mm between wt and *Gli3^xt/xt^* embryos (n = 6 each) using the student’s t test. The breadth of the band of *Tbx3* expression was measured (in mm) at the location of mammary placode 3 (that falls between somites 16 and 17 in wt embryos [54]) and compared between wt and *Gli3^xt/xt^* embryos (n = 6 each) using the student’s t test.

### Carmine Staining

For detection of mammary sprouts in E18 embryos, skins were removed from the embryos and fixed in 4% PFA for 1 hour. The skins were washed in PBS then stained for 1 hour in carmine solution diluted 1∶5 in water. Carmine was prepared by boiling 1 g carmine alum and 25 g aluminum potassium sulfate in 500 mL of water for 20 minutes followed by filtration.

### Histology

For histological analysis, embryos and tissues were stained as above with X-Gal, post-fixed with 4% PFA overnight at 4°C then embedded in paraffin and sectioned. Isopropanol was substituted for xylene to prevent diffusion of the X-Gal stain during processing.

### Immunohistochemistry

Four µm sections were deparaffinized by baking at 60°C and incubating slides in Citrisolv (Fisher Scientific, Pittsburgh, PA) and rehydrated through a graded series of ethanol. Citric acid antigen retrieval was performed for all antibodies by placing slides in 10 mM sodium citrate pH 6.0 and boiling in a microwave at 90 W power for 30 minutes. Primary rabbit antibodies against AR (Santa Cruz Biotechnologies, Santa Cruz, CA) (1∶100), Desmin (Abcam, Cambridge, MA) (1∶50), Peripherin (Chemicon, Temecula CA) (1∶1000) and Von Willebrand Factor (Sigma Aldrich) (1∶1000), mouse antibodies against p63 (Neomarkers, Freemont, CA) (1∶500), SMA (Sigma Aldrich) (1∶5000), Tenascin C (Immuno Biological Laboratories, Gunma, Japan) (1∶500) and ER (Novocastra, Newcastle, U.K.) (1∶500) and guinea pig antibodies against Vimentin (Progen, Heidelberg, Germany) (1∶1000) were added overnight at 4°C. Biotin-labeled secondary antibodies (Vector Laboratories, Burlingame, CA) (1∶1000) and streptavidin-HRP (Vector Laboratories) (1∶200) were added for 30 minutes each, and colorimetrically detected using diaminobenzidine (Vector Laboratories). Sections were counterstained for better visualization in 0.1% solution of Nuclear Fast Red (NFR, Polyscientific, Bayshore, NY) for 1 minute and washed in a stream of running water for 5 minutes. Sections were then dehydrated and dipped in Citrisolv (Fisher Scientific) before being mounted in Cytoseal (VWR, Radnor, PA).

## References

[1] Veltmaat JM, Mailleux AA, Thiery JP, Bellusci S (2003) Mouse embryonic mammogenesis as a model for the molecular regulation of pattern formation. Differentiation 71: 1–17. 12558599 10.1046/j.1432-0436.2003.700601.x

[2] Veltmaat JM, Van Veelen W, Thiery JP, Bellusci S (2004) Identification of the mammary line in mouse by Wnt10b expression. Dev Dyn 229: 349–356. 14745960 10.1002/dvdy.10441

[3] Cowin P, Wysolmerski J (2010) Molecular mechanisms guiding embryonic mammary gland development. Cold Spring Harbor perspectives in biology 2: a003251. 20484386 10.1101/cshperspect.a003251 PMC2869520

[4] Lee MY, Racine V, Jagadpramana P, Sun L, Yu W, et al (2011) Ectodermal influx and cell hypertrophy provide early growth for all murine mammary rudiments, and are differentially regulated among them by Gli3. PLoS One 6: e26242. 22046263 10.1371/journal.pone.0026242 PMC3203106

[5] van Genderen C, Okamura RM, Farinas I, Quo R-G, Parslow TG, et al (1994) Development of several organs that require inductive epithelial-mesenchymal interactions is impaired in Lef-1 deficient mice. Genes and Development 8: 2691–2704. 7958926 10.1101/gad.8.22.2691

[6] Mailleux AA, Spencer-Dene B, Dillon C, Ndiaye D, Savona-Baron C, et al (2002) Role of FGF10/FGFR2b signaling during mammary gland development in the mouse embryo. Development 129: 53–60. 11782400 10.1242/dev.129.1.53

[7] Davenport TG, Jerome-Majewska LA, Papaioannou VE (2003) Mammary gland, limb and yolk sac defects in mice lacking Tbx3, the gene mutated in human ulnar mammary syndrome. Development 130: 2263–2273. 12668638 10.1242/dev.00431

[8] Howard B, Panchal H, McCarthy A, Ashworth A (2005) Identification of the scaramanga gene implicates Neuregulin3 in mammary gland specification. Genes Dev 19: 2078–2090. 16140987 10.1101/gad.338505 PMC1199577

[9] Robinson GW (2007) Cooperation of signalling pathways in embryonic mammary gland development. Nat Rev Genet 8: 963–972. 18007652 10.1038/nrg2227

[10] Cho KW, Kwon HJ, Shin JO, Lee JM, Cho SW, et al (2012) Retinoic acid signaling and the initiation of mammary gland development. Dev Biol 365: 259–266. 22387209 10.1016/j.ydbio.2012.02.020

[11] Hens JR, Dann P, Zhang JP, Harris S, Robinson GW, et al (2007) BMP4 and PTHrP interact to stimulate ductal outgrowth during embryonic mammary development and to inhibit hair follicle induction. Development 134: 1221–1230. 17301089 10.1242/dev.000182

[12] Cho KW, Kim JY, Song SJ, Farrell E, Eblaghie MC, et al (2006) Molecular interactions between Tbx3 and Bmp4 and a model for dorsoventral positioning of mammary gland development. Proc Natl Acad Sci U S A 103: 16788–16793. 17071745 10.1073/pnas.0604645103 PMC1636533

[13] Wysolmerski JJ, Philbrick WM, Dunbar ME, Lanske B, Kronenberg H, et al (1998) Rescue of the parathyroid hormone-related protein knockout mouse demonstrates that parathyroid hormone-related protein is essential for mammary gland development. Development 125: 1285–1294. 9477327 10.1242/dev.125.7.1285

[14] Foley J, Dann P, Hong J, Cosgrove J, Dreyer B, et al (2001) Parathyroid hormone-related protein maintains mammary epithelial fate and triggers nipple skin differentiation during embryonic breast development. Development 128: 513–525. 11171335 10.1242/dev.128.4.513

[15] Dunbar ME, Dann PR, Robinson GW, Hennighausen L, Zhang JP, et al (1999) Parathyroid hormone-related protein signaling is necessary for sexual dimorphism during embryonic mammary development. Development 126: 3485–3493. 10409496 10.1242/dev.126.16.3485

[16] Mayer JA, Foley J, De La Cruz D, Chuong CM, Widelitz R (2008) Conversion of the nipple to hair-bearing epithelia by lowering bone morphogenetic protein pathway activity at the dermal-epidermal interface. Am J Pathol 173: 1339–1348. 18832580 10.2353/ajpath.2008.070920 PMC2570124

[17] Kratochwil K (1971) In vitro analysis of the hormonal basis for the sexual dimorphism in the embryonic development of the mouse mammary gland. J Embryol Exp Morphol 25: 141–153. 5548210

[18] Kratochwil K (1977) Development and loss of androgen responsiveness in the embryonic rudiment of the mouse mammary gland. Dev Biol 61: 358–365. 590632 10.1016/0012-1606(77)90305-0

[19] Kratochwil K, Schwartz P (1976) Tissue interaction in androgen response of embryonic mammary rudiment of mouse: identification of target tissue for testosterone. Proc Natl Acad Sci U S A 73: 4041–4044. 1069291 10.1073/pnas.73.11.4041 PMC431320

[20] Heuberger B, Fitzka I, Wasner G, Kratochwil K (1982) Induction of androgen receptor formation by epithelium-mesenchyme interaction in embryonic mouse mammary gland. Proc Natl Acad Sci U S A 79: 2957–2961. 6953441 10.1073/pnas.79.9.2957 PMC346327

[21] St-Jacques B, Dassule HR, Karavanova I, Botchkarev VA, Li J, et al (1998) Sonic hedgehog signaling is essential for hair development. Curr Biol 8: 1058–1068. 9768360 10.1016/s0960-9822(98)70443-9

[22] Chiang C, Swan RZ, Grachtchouk M, Bolinger M, Litingtung Y, et al (1999) Essential role for Sonic hedgehog during hair follicle morphogenesis. Dev Biol 205: 1–9. 9882493 10.1006/dbio.1998.9103

[23] McMahon AP, Ingham PW, Tabin CJ (2003) Developmental roles and clinical significance of hedgehog signaling. Curr Top Dev Biol 53: 1–114. 12509125 10.1016/s0070-2153(03)53002-2

[24] Mill P, Mo R, Fu H, Grachtchouk M, Kim PC, et al (2003) Sonic hedgehog-dependent activation of Gli2 is essential for embryonic hair follicle development. Genes Dev 17: 282–294. 12533516 10.1101/gad.1038103 PMC195973

[25] Cobourne MT, Miletich I, Sharpe PT (2004) Restriction of sonic hedgehog signalling during early tooth development. Development 131: 2875–2885. 15151988 10.1242/dev.01163

[26] Riobo NA, Manning DR (2007) Pathways of signal transduction employed by vertebrate Hedgehogs. Biochem J 403: 369–379. 17419683 10.1042/BJ20061723

[27] Jiang J, Hui CC (2008) Hedgehog signaling in development and cancer. Dev Cell 15: 801–812. 19081070 10.1016/j.devcel.2008.11.010 PMC6443374

[28] Hui CC, Slusarski D, Platt KA, Holmgren R, Joyner AL (1994) Expression of three mouse homologs of the Drosophila segment polarity gene cubitus interruptus, Gli, Gli-2, and Gli-3, in ectoderm- and mesoderm-derived tissues suggests multiple roles during postimplantation development. Dev Biol 162: 402–413. 8150204 10.1006/dbio.1994.1097

[29] Wang Y, McMahon AP, Allen BL (2007) Shifting paradigms in Hedgehog signaling. Curr Opin Cell Biol 19: 159–165. 17303409 10.1016/j.ceb.2007.02.005

[30] Tenzen T, Allen BL, Cole F, Kang JS, Krauss RS, et al (2006) The cell surface membrane proteins Cdo and Boc are components and targets of the Hedgehog signaling pathway and feedback network in mice. Dev Cell 10: 647–656. 16647304 10.1016/j.devcel.2006.04.004

[31] Bai CB, Stephen D, Joyner AL (2004) All mouse ventral spinal cord patterning by hedgehog is Gli dependent and involves an activator function of Gli3. Dev Cell 6: 103–115. 14723851 10.1016/s1534-5807(03)00394-0

[32] Blaess S, Corrales JD, Joyner AL (2006) Sonic hedgehog regulates Gli activator and repressor functions with spatial and temporal precision in the mid/hindbrain region. Development 133: 1799–1809. 16571630 10.1242/dev.02339

[33] Bok J, Dolson DK, Hill P, Ruther U, Epstein DJ, et al (2007) Opposing gradients of Gli repressor and activators mediate Shh signaling along the dorsoventral axis of the inner ear. Development 134: 1713–1722. 17395647 10.1242/dev.000760

[34] Sasaki H, Nishizaki Y, Hui C, Nakafuku M, Kondoh H (1999) Regulation of Gli2 and Gli3 activities by an amino-terminal repression domain: implication of Gli2 and Gli3 as primary mediators of Shh signaling. Development 126: 3915–3924. 10433919 10.1242/dev.126.17.3915

[35] Aza-Blanc P, Lin HY, Ruiz i Altaba A, Kornberg TB (2000) Expression of the vertebrate Gli proteins in Drosophila reveals a distribution of activator and repressor activities. Development 127: 4293–4301. 10976059 10.1242/dev.127.19.4293

[36] Bai CB, Joyner AL (2001) Gli1 can rescue the in vivo function of Gli2. Development 128: 5161–5172. 11748151 10.1242/dev.128.24.5161

[37] Bai CB, Auerbach W, Lee JS, Stephen D, Joyner AL (2002) Gli2, but not Gli1, is required for initial Shh signaling and ectopic activation of the Shh pathway. Development 129: 4753–4761. 12361967 10.1242/dev.129.20.4753

[38] Hynes M, Stone DM, Dowd M, Pitts-Meek S, Goddard A, et al (1997) Control of cell pattern in the neural tube by the zinc finger transcription factor and oncogene Gli-1. Neuron 19: 15–26. 9247260 10.1016/s0896-6273(00)80344-x

[39] Lee Y-S, Dlugosz AA, McKay R, Dean NM, Yuspa SH (1997) Definition by Specific Antisense Oligonucleotides of a Role fro Protein Kinase C-alpha in Expression of Differentiation Markers in Normal and Neoplastic Mouse Epidermal Keratinocytes. Molecular Carcinogenesis 18: 44–53. 9022812

[40] Dai P, Akimaru H, Tanaka Y, Maekawa T, Nakafuku M, et al (1999) Sonic Hedgehog-induced activation of the Gli1 promoter is mediated by GLI3. J Biol Chem 274: 8143–8152. 10075717 10.1074/jbc.274.12.8143

[41] Park HL, Bai C, Platt KA, Matise MP, Beeghly A, et al (2000) Mouse Gli1 mutants are viable but have defects in SHH signaling in combination with a Gli2 mutation. Development 127: 1593–1605. 10725236 10.1242/dev.127.8.1593

[42] Lee J, Platt KA, Censullo P, Ruiz i Altaba A (1997) Gli1 is a target of Sonic hedgehog that induces ventral neural tube development. Development 124: 2537–2552. 9216996 10.1242/dev.124.13.2537

[43] Wang B, Fallon JF, Beachy PA (2000) Hedgehog-regulated processing of Gli3 produces an anterior/posterior repressor gradient in the developing vertebrate limb. Cell 100: 423–434. 10693759 10.1016/s0092-8674(00)80678-9

[44] Li Y, Zhang H, Choi SC, Litingtung Y, Chiang C (2004) Sonic hedgehog signaling regulates Gli3 processing, mesenchymal proliferation, and differentiation during mouse lung organogenesis. Dev Biol 270: 214–231. 15136151 10.1016/j.ydbio.2004.03.009

[45] Marigo V, Johnson RL, Vortkamp A, Tabin CJ (1996) Sonic hedgehog differentially regulates expression of GLI and GLI3 during limb development. Dev Biol 180: 273–283. 8948590 10.1006/dbio.1996.0300

[46] Hui CC, Angers S (2011) Gli proteins in development and disease. Annu Rev Cell Dev Biol 27: 513–537. 21801010 10.1146/annurev-cellbio-092910-154048

[47] Ishikawa H, Marshall WF (2011) Ciliogenesis: building the cell’s antenna. Nat Rev Mol Cell Biol 12: 222–234. 21427764 10.1038/nrm3085

[48] Johnson ET, Nicola T, Roarty K, Yoder BK, Haycraft CJ, et al (2008) Role for primary cilia in the regulation of mouse ovarian function. Dev Dyn 237: 2053–2060. 18629867 10.1002/dvdy.21612

[49] Chuong CM, Patel N, Lin J, Jung HS, Widelitz RB (2000) Sonic hedgehog signaling pathway in vertebrate epithelial appendage morphogenesis: perspectives in development and evolution. Cell Mol Life Sci 57: 1672–1681. 11130174 10.1007/PL00000650 PMC4381998

[50] Michno K, Boras-Granic K, Mill P, Hui CC, Hamel PA (2003) Shh expression is required for embryonic hair follicle but not mammary gland development. Dev Biol 264: 153–165. 14623238 10.1016/s0012-1606(03)00401-9

[51] Hatsell SJ, Cowin P (2006) Gli3-mediated repression of Hedgehog targets is required for normal mammary development. Development 133: 3661–3670. 16914490 10.1242/dev.02542

[52] Gritli-Linde A, Hallberg K, Harfe BD, Reyahi A, Kannius-Janson M, et al (2007) Abnormal hair development and apparent follicular transformation to mammary gland in the absence of hedgehog signaling. Dev Cell 12: 99–112. 17199044 10.1016/j.devcel.2006.12.006 PMC1885956

[53] Widelitz RB, Veltmaat JM, Mayer JA, Foley J, Chuong CM (2007) Mammary glands and feathers: comparing two skin appendages which help define novel classes during vertebrate evolution. Semin Cell Dev Biol 18: 255–266. 17382566 10.1016/j.semcdb.2007.02.005 PMC4382004

[54] Veltmaat JM, Relaix F, Le LT, Kratochwil K, Sala FG, et al (2006) Gli3-mediated somitic Fgf10 expression gradients are required for the induction and patterning of mammary epithelium along the embryonic axes. Development 133: 2325–2335. 16720875 10.1242/dev.02394

[55] Pu Y, Huang L, Prins GS (2004) Sonic hedgehog-patched Gli signaling in the developing rat prostate gland: lobe-specific suppression by neonatal estrogens reduces ductal growth and branching. Dev Biol 273: 257–275. 15328011 10.1016/j.ydbio.2004.06.002 PMC2978068

[56] Madison BB, Braunstein K, Kuizon E, Portman K, Qiao XT, et al (2005) Epithelial hedgehog signals pattern the intestinal crypt-villus axis. Development 132: 279–289. 15590741 10.1242/dev.01576

[57] Jho EH, Zhang T, Domon C, Joo CK, Freund JN, et al (2002) Wnt/beta-catenin/Tcf signaling induces the transcription of Axin2, a negative regulator of the signaling pathway. Mol Cell Biol 22: 1172–1183. 11809808 10.1128/MCB.22.4.1172-1183.2002 PMC134648

[58] Lustig B, Jerchow B, Sachs M, Weiler S, Pietsch T, et al (2002) Negative feedback loop of Wnt signaling through upregulation of conductin/axin2 in colorectal and liver tumors. Mol Cell Biol 22: 1184–1193. 11809809 10.1128/MCB.22.4.1184-1193.2002 PMC134640

[59] Hiremath M, Dann P, Fischer J, Butterworth D, Boras-Granic K, et al (2012) Parathyroid hormone-related protein activates Wnt signaling to specify the embryonic mammary mesenchyme. Development 139: 4239–4249. 23034629 10.1242/dev.080671 PMC3478689

[60] Eblaghie MC, Song SJ, Kim JY, Akita K, Tickle C, et al (2004) Interactions between FGF and Wnt signals and Tbx3 gene expression in mammary gland initiation in mouse embryos. J Anat 205: 1–13. 15255957 10.1111/j.0021-8782.2004.00309.x PMC1571327

[61] Jerome-Majewska LA, Jenkins GP, Ernstoff E, Zindy F, Sherr CJ, et al (2005) Tbx3, the ulnar-mammary syndrome gene, and Tbx2 interact in mammary gland development through a p19Arf/p53-independent pathway. Dev Dyn 234: 922–933. 16222716 10.1002/dvdy.20575

[62] Liu J, Esmailpour T, Shang X, Gulsen G, Liu A, et al (2011) TBX3 over-expression causes mammary gland hyperplasia and increases mammary stem-like cells in an inducible transgenic mouse model. BMC Dev Biol 11: 65. 22039763 10.1186/1471-213X-11-65 PMC3217876

[63] Bamshad M, Lin RC, Law DJ, Watkins WC, Krakowiak PA, et al (1997) Mutations in human TBX3 alter limb, apocrine and genital development in ulnar-mammary syndrome. Nat Genet 16: 311–315. 9207801 10.1038/ng0797-311

[64] Network CGA (2012) Comprehensive molecular portraits of human breast tumours. Nature 490: 61–70. 23000897 10.1038/nature11412 PMC3465532

[65] Rowley M, Grothey E, Couch FJ (2004) The role of Tbx2 and Tbx3 in mammary development and tumorigenesis. J Mammary Gland Biol Neoplasia 9: 109–118. 15300007 10.1023/B:JOMG.0000037156.64331.3f

[66] Aubele M, Auer G, Braselmann H, Nahrig J, Zitzelsberger H, et al (2002) Chromosomal imbalances are associated with metastasis-free survival in breast cancer patients. Anal Cell Pathol 24: 77–87. 12446957 10.1155/2002/820269 PMC4618589

[67] Fan W, Huang X, Chen C, Gray J, Huang T (2004) TBX3 and its isoform TBX3+2a are functionally distinctive in inhibition of senescence and are overexpressed in a subset of breast cancer cell lines. Cancer Res 64: 5132–5139. 15289316 10.1158/0008-5472.CAN-04-0615

[68] Yarosh W, Barrientos T, Esmailpour T, Lin L, Carpenter PM, et al (2008) TBX3 is overexpressed in breast cancer and represses p14 ARF by interacting with histone deacetylases. Cancer Res 68: 693–699. 18245468 10.1158/0008-5472.CAN-07-5012

[69] Yang L, Cai CL, Lin L, Qyang Y, Chung C, et al (2006) Isl1Cre reveals a common Bmp pathway in heart and limb development. Development 133: 1575–1585. 16556916 10.1242/dev.02322 PMC5576437

[70] Behesti H, Holt JK, Sowden JC (2006) The level of BMP4 signaling is critical for the regulation of distinct T-box gene expression domains and growth along the dorso-ventral axis of the optic cup. BMC Dev Biol 6: 62. 17173667 10.1186/1471-213X-6-62 PMC1764729

[71] Eriksson KS, Mignot E (2009) T-box 3 is expressed in the adult mouse hypothalamus and medulla. Brain Res 1302: 233–239. 19765559 10.1016/j.brainres.2009.08.101 PMC2805014

[72] Roberts DJ, Johnson RL, Burke AC, Nelson CE, Morgan BA, et al (1995) Sonic hedgehog is an endodermal signal inducing Bmp-4 and Hox genes during induction and regionalization of the chick hindgut. Development 121: 3163–3174. 7588051 10.1242/dev.121.10.3163

[73] Sukegawa A, Narita T, Kameda T, Saitoh K, Nohno T, et al (2000) The concentric structure of the developing gut is regulated by Sonic hedgehog derived from endodermal epithelium. Development 127: 1971–1980. 10751185 10.1242/dev.127.9.1971

[74] Yu J, Carroll TJ, McMahon AP (2002) Sonic hedgehog regulates proliferation and differentiation of mesenchymal cells in the mouse metanephric kidney. Development 129: 5301–5312. 12399320 10.1242/dev.129.22.5301

[75] Kawai S, Sugiura T (2001) Characterization of human bone morphogenetic protein (BMP)-4 and -7 gene promoters: activation of BMP promoters by Gli, a sonic hedgehog mediator. Bone 29: 54–61. 11472891 10.1016/s8756-3282(01)00470-7

[76] Cartharius K, Frech K, Grote K, Klocke B, Haltmeier M, et al (2005) MatInspector and beyond: promoter analysis based on transcription factor binding sites. Bioinformatics 21: 2933–2942. 15860560 10.1093/bioinformatics/bti473

[77] Hens JR, Wysolmerski JJ (2005) Key stages of mammary gland development: molecular mechanisms involved in the formation of the embryonic mammary gland. Breast Cancer Res 7: 220–224. 16168142 10.1186/bcr1306 PMC1242158

[78] Dunbar ME, Young P, Zhang JP, McCaughern-Carucci J, Lanske B, et al (1998) Stromal cells are critical targets in the regulation of mammary ductal morphogenesis by parathyroid hormone-related protein. Dev Biol 203: 75–89. 9806774 10.1006/dbio.1998.9029

[79] Dunbar ME, Wysolmerski JJ (1999) Parathyroid hormone-related protein: a developmental regulatory molecule necessary for mammary gland development. J Mammary Gland Biol Neoplasia 4: 21–34. 10219904 10.1023/a:1018700502518

[80] Boras-Granic K, Chang H, Grosschedl R, Hamel PA (2006) Lef1 is required for the transition of Wnt signaling from mesenchymal to epithelial cells in the mouse embryonic mammary gland. Dev Biol 295: 219–231. 16678815 10.1016/j.ydbio.2006.03.030

[81] Chu EY, Hens J, Andl T, Kairo A, Yamaguchi TP, et al (2004) Canonical WNT signaling promotes mammary placode development and is essential for initiation of mammary gland morphogenesis. Development 131: 4819–4829. 15342465 10.1242/dev.01347

[82] Abdalkhani A, Sellers R, Gent J, Wulitich H, Childress S, et al (2002) Nipple connective tissue and its development: insights from the K14-PTHrP mouse. Mech Dev 115: 63–77. 12049768 10.1016/s0925-4773(02)00092-8

[83] Hill P, Gotz K, Ruther U (2009) A SHH-independent regulation of Gli3 is a significant determinant of anteroposterior patterning of the limb bud. Dev Biol 328: 506–516. 19248778 10.1016/j.ydbio.2009.02.017

[84] McGlinn E, van Bueren KL, Fiorenza S, Mo R, Poh AM, et al (2005) Pax9 and Jagged1 act downstream of Gli3 in vertebrate limb development. Mech Dev 122: 1218–1233. 16169709 10.1016/j.mod.2005.06.012

[85] McDermott A, Gustafsson M, Elsam T, Hui CC, Emerson CP Jr, et al (2005) Gli2 and Gli3 have redundant and context-dependent function in skeletal muscle formation. Development 132: 345–357. 15604102 10.1242/dev.01537

[86] Methot N, Basler K (1999) Hedgehog controls limb development by regulating the activities of distinct transcriptional activator and repressor forms of Cubitus interruptus. Cell 96: 819–831. 10102270 10.1016/s0092-8674(00)80592-9

[87] Motoyama J, Takabatake T, Takeshima K, Hui C (1998) Ptch2, a second mouse Patched gene is co-expressed with Sonic hedgehog. Nat Genet 18: 104–106. 9462734 10.1038/ng0298-104

[88] Hatsell S, Frost AR (2007) Hedgehog signaling in mammary gland development and breast cancer. J Mammary Gland Biol Neoplasia 12: 163–173. 17623270 10.1007/s10911-007-9048-2

[89] Kobayashi T, Kronenberg HM, Foley J (2005) Reduced expression of the PTH/PTHrP receptor during development of the mammary gland influences the function of the nipple during lactation. Dev Dyn 233: 794–803. 15880431 10.1002/dvdy.20406

[90] Mo R, Freer AM, Zinyk DL, Crackower MA, Michaud J, et al (1997) Specific and redundant functions of Gli2 and Gli3 zinc finger genes in skeletal patterning and development. Development 124: 113–123. 9006072 10.1242/dev.124.1.113

[91] Wysolmerski JJ, Broadus AE, Zhou J, Fuchs E, Milstone LM, et al (1994) Overexpression of parathyroid hormone-related protein in the skin of transgenic mice interferes with hair follicle development. Proc Natl Acad Sci U S A 91: 1133–1137. 7508121 10.1073/pnas.91.3.1133 PMC521468

